# Impact of Health Literacy on Patient-Reported Outcomes in Benign Gynecology: A Systematic Review

**DOI:** 10.7759/cureus.58661

**Published:** 2024-04-20

**Authors:** Ashmita Singh, Emma Skolnik, Elizabeth Miazga, Alysha Nensi

**Affiliations:** 1 General Surgery, Northern Ontario School of Medicine, Sudbury, CAN; 2 Obstetrics and Gynecology, University of Toronto, Toronto, CAN; 3 Obstetrics and Gynecology, Trillium Health Partners - Credit Valley Hospital, Mississauga, CAN; 4 Obstetrics and Gynecology, St. Michael's Hospital, Toronto, CAN

**Keywords:** health literacy, general gynecology, healthcare outcomes, patient-reported outcome, benign gynecology

## Abstract

The objective of this study was to systematically review the relationship between low health literacy and patient-reported outcomes in patients with benign gynecologic conditions. In this specific population, we also sought to determine the current reported prevalence of low health literacy, examine demographic characteristics that may be related to low health literacy, and collate any health literacy interventions described in the literature. A systematic search of MEDLINE (Medical Literature Analysis and Retrieval System Online), Embase, The Cochrane Library, Web of Science, PubMed, and clinicaltrials.gov was performed on July 12, 2021, and repeated on October 13, 2023, for terms related to health literacy, specific health literacy measures, and benign gynecologic conditions. There were language or publication period restrictions. Inclusion required primary literature to report associations between health literacy and patient-reported outcomes, using validated tools to quantitatively measure each, in adult women with benign gynecologic conditions. Title screening, abstract screening, and full-text review were conducted with Covidence software (Melbourne, Australia) assisting with the review process. Of the 18,701 studies returned using our search strategy, 25 were selected for full-text review. Of these, no studies met inclusion criteria and reported an association between health literacy and patient-reported outcomes. This study identified a large gap in the literature. Future work should be directed at evaluating the association between health literacy and patient-reported outcomes in benign gynecology to inform patient-centered interventions and care provision.

## Introduction and background

Health literacy has been defined as “the extent to which individuals have the capacity to obtain, process, and understand basic health information that is needed to make appropriate health decisions” [1]. Poor or inadequate health literacy has been linked to negative patient outcomes such as inferior disease-specific knowledge [2], reduced self-reported health [3], increased hospitalizations and readmissions [4,5], and higher mortality in older adults [6]. Low levels of health literacy are more prevalent in minority populations, including persons for whom English is not their first language, people with low levels of income and education, people with compromised health status, and elderly communities [7]. 

Given the significant burden of inadequate health literacy on the health of people, especially within marginalized populations, it is imperative that healthcare providers are aware of the direct health implications, and patient-centered outcomes within specific clinical contexts. Patient-reported outcomes reflect the status of a patient’s health condition and are obtained from the independent perspective of a patient [8]. Patient-reported outcomes measures are a unique and high-quality patient-centered evaluation tool, as they are derived from a patient’s beliefs regarding their individual health status and needs without secondary interpretation from others.

The management of benign gynecologic disorders, such as abnormal uterine bleeding, fibroids, endometriosis, and pelvic organ prolapse, often requires advanced health literacy skills. This is due to the importance of understanding relevant anatomy and clinical presentations, recognizing the severity of associated symptoms, managing medication regimens, and comprehending the available treatment options, both medical and surgical. For example, the inability to understand the nuanced instructions involving medication dosing or pre-operative directions can result in compromised patient safety. In this way, low health literacy can negatively impact the delivery of equitable patient-centered care in gynecology. 

While some studies have looked at the general impact of health literacy on health outcomes, most notably the work by Berkman and colleagues [7], and at least one systematic review has considered the role of health literacy in the reproductive health of women [9], we hypothesized there is a paucity of work specifically focusing on patients seeking treatment for benign gynecologic conditions. We aimed to systematically review existing data regarding health literacy and patient-driven health outcomes in this population, with the primary objective of evaluating the relationship between health literacy and patient-reported outcomes in patients with benign gynecologic conditions. Secondarily, we also sought to determine the current reported prevalence of inadequate health literacy in patients with benign gynecological conditions, examine patient and demographic characteristics that may be significantly associated with low health literacy in this population, and collate designs and results of any interventions described in the literature which reduced literacy-related disparities in this population.

## Review

Methods

The study protocol was registered with the International Prospective Register of Systematic Reviews (PROSPERO) (registration number: CRD42021261924) on July 30, 2021.

Sources

MEDLINE (Medical Literature Analysis and Retrieval System Online), Embase, The Cochrane Library (Cochrane Database of Systematic Reviews, Cochrane Central Register of Controlled Trials (CENTRAL), Cochrane Methodology Register), Web of Science, PubMed (for items not on MEDLINE), and clinicaltrials.gov databases were searched on July 12, 2021, and again on October 13, 2023, for terms related to health literacy, specific health literacy measures, and benign gynecologic conditions (abnormal uterine bleeding, endometriosis, uterine fibroids, benign ovarian masses, and pelvic organ prolapse). Specific search strategies and search terms are detailed in the Appendices. These were developed in keeping with strategies used in other systematic reviews on these topics [7,10-14]. There were no language or publication period restrictions. 

Study Selection

Articles were assessed for inclusion based on the following criteria: (i) population of adult women over the age of 18 with benign gynecologic conditions, (ii) use of validated tools to quantitatively measure health literacy and patient-reported outcomes, (iii) evidence of analyzing and reporting associations between health literacy and patient-reported outcomes, (iv) peer-reviewed primary literature. For the purpose of this review, benign gynecologic disorders were defined as: abnormal uterine bleeding, uterine fibroids, endometriosis and menstrual disorders, benign ovarian masses, and pelvic organ prolapse. 

There were no restrictions on the number of participants, study setting (primary, secondary, or tertiary care), country, or length of follow-up. Studies of any design, except for case reports, were considered for inclusion. Studies were also excluded from the review if they were non-peer-reviewed, grey literature, or not considered primary research, and if the prevalence of adequate and inadequate health literacy in their study sample was not reported or discernable from the study. 

Study selection occurred as a three-stage screening process using Covidence software (Melbourne, Australia); this included a title screen, an abstract screen, and a full-text review. First, a review of titles was conducted to remove any ineligible studies. Following this, an abstract screen and subsequent full-text review for the remaining articles were undertaken by two independent reviewers (AS, ES). Reviewers were blinded to one another’s decisions during the abstract and full-text screen; any disagreements regarding the inclusion of an article were resolved by a third reviewer (EM). Prior to the study selection process, all reviewers completed a pilot screen with 10 articles to ensure a full and clear understanding of the inclusion and exclusion criteria. 

Results

**Figure 1:**
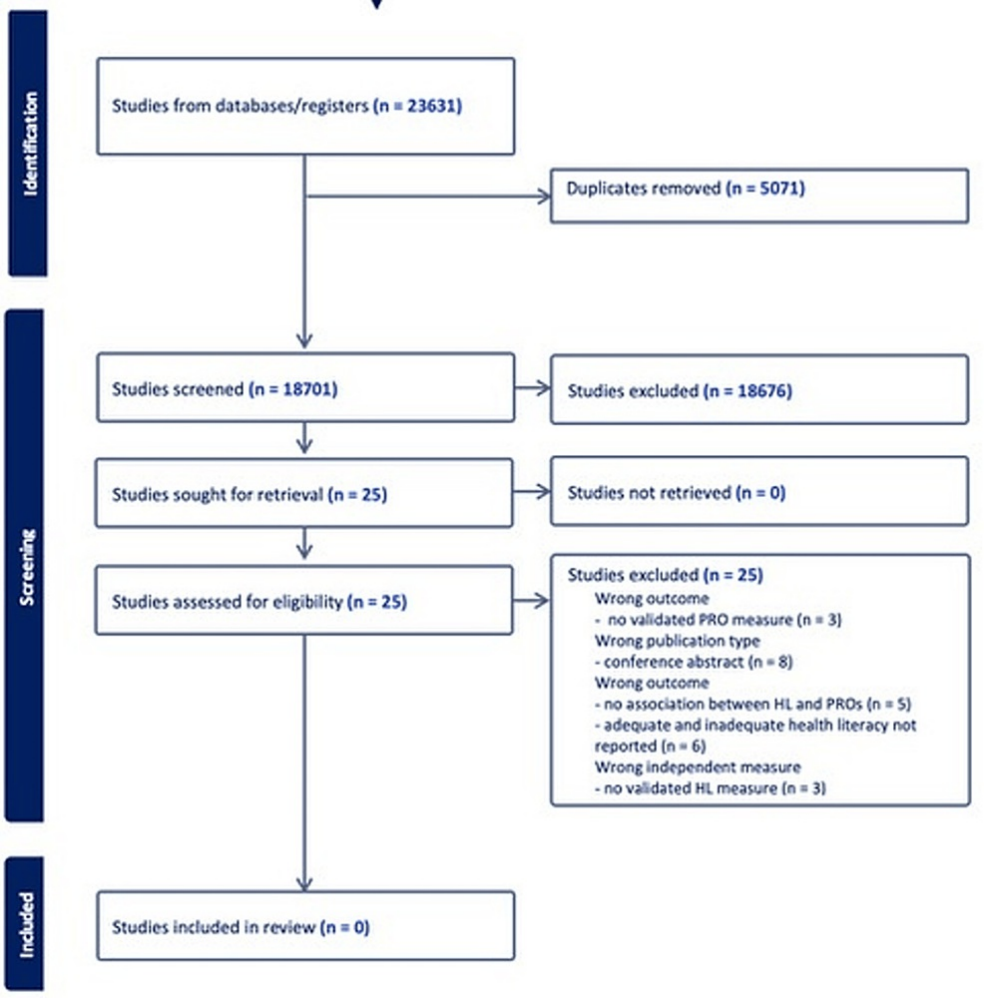
PRISMA flow diagram of study selection PRISMA: Preferred Reporting Items for Systematic Reviews and Meta-Analyses; HL: health literacy; PRO: patient-reported outcome

Figure 1 shows the Preferred Reporting Items for Systematic Reviews and Meta-Analyses (PRISMA) flow chart detailing the selection process. Overall, after the removal of duplicates, 18,560 articles were returned through the search. Another 141 studies were identified by searching Clinicaltrials.gov. Following the title review, 926 studies proceeded to the abstract review with 25 studies being selected for full-text review. None of the 25 articles fulfilled the inclusion criteria, and thus, could not be included in the review. Given this, manual reference mining was not conducted as there were zero included articles. Reasons for exclusion included: wrong publication type (non-peer reviewed, conference abstracts), lack of validated health literacy measures, and wrong study outcomes. 

Discussion

Although existing literature was broadly searched over many databases, we did not find any existing studies that investigated the role of health literacy and its impact on patient-reported outcomes among patients with benign gynecologic conditions. Of the studies that were included in the full-text review, those relating to health literacy, patient-reported measures, and knowledge have been summarized in Table 1 [15-33], including major findings, and reasons for exclusion in order to highlight what the current state of published literature is in this domain.

**Table 1:** Summary of the relevant articles noted during the full text screen, organized by key themes identified TOFHLA: Test of Functional Health Literacy in Adults; HL: health literacy; PRO: patient-reported outcome; REALM-R: Rapid Estimate of Adult Literacy in Medicine – Revised

Study	Key findings	Reason for exclusion
Health literacy and knowledge		
Anger et al., 2012 [15]	Health literacy did not correlate with understanding and knowledge of pelvic floor disease. Health literacy decreased with increasing age. Health literacy measured using TOFHLA.	No association between HL and PRO
Brocks et al., 2013 [27]	Inadequate health literacy in 13% of the study sample (Urban African American women). Adequate health literacy correlated with increased knowledge of abnormal uterine bleeding. Health literacy measured using validated single-question Likert scale.	Conference abstract
Ecpo et al., 2014 [16]	Inadequate health literacy in 13% of the study sample (African American women). Health literacy was not correlated with uterine fibroid knowledge. Health literacy measured using validated single-question Likert scale.	No association between HL and PRO
Knowledge and satisfaction (patient-reported experience measures)		
Solberg et al., 2010 [28]	Increased knowledge associated with increased satisfaction with decision related to uterine fibroid management, following use of a decision aid.	Adequate and inadequate HL not reported
Pallett et al., 2016 [29]	Addition of video counselling increased comprehension of hysterectomy consent form and led to greater patient satisfaction. health literacy measured using REALM score.	No PRO measure
Murray, 2020 [17]	Higher subjective knowledge associated with increased patient satisfaction with clinical consults in urogynecology. Health literacy, measured with REALM-R, not correlated with patient satisfaction.	Conference abstract
al Kurdi et al., 2021 [30]	Improved knowledge and service satisfaction in patients with PCOS following an educational intervention.	No association between HL and PRO
Knowledge and PROs		
Geoffrion et al., 2008 [31]	Knowledge of pelvic floor exercises and symptoms of pelvic floor disease improved following intervention of an educational workshop.	Conference abstract
Geoffrion et al., 2009 [32]	Increased knowledge of pelvic organ prolapse and increased quality of life outcomes following implementation of educational program intervention.	Adequate and inadequate health literacy not reported
Goodridge et al., 2021 [33]	Increased knowledge of pelvic floor disorders is correlated to better patient-reported outcomes and quality of life measures	No validated HL measure

Specifically, three recurrent themes emerged during the full-text review: (i) Studies that investigated the relationship between health literacy and knowledge of benign gynecologic conditions in specific populations, (ii) Studies describing the impact of knowledge and/or health literacy on patient satisfaction and patient experience, as opposed to patient-reported outcomes, and (iii) Studies that explored the relationship between patient-reported outcomes and knowledge. Although numerous studies on benign gynecologic conditions briefly mention health literacy with regard to patient outcomes or experiences, it was evident while conducting our full-text review that health literacy has only been superficially explored in this population, and has not been included as an important predictor and often not measured using validated tools. Despite being mentioned in studies, it was often not reported and was simply said to have been measured as a way of minimizing experimental- or cohort-group differences. Of those that did measure health literacy, there was an acknowledgment of its importance but a failure to proceed with meaningful reporting or analysis.

We noted that patient knowledge, regarding specific benign gynecologic conditions was used in many studies as an independent variable, as opposed to health literacy, with regards to impact on patient outcomes. Moreover, some of these studies found that health literacy did not correlate to specific disease knowledge or to patient satisfaction [15-17] (Table 1). These results draw attention to the importance of understanding whether knowledge or health literacy is a more effective tool in improving patient outcomes. Health literacy has been found to be independently related to disease knowledge of many chronic diseases, including asthma, diabetes, congestive heart failure, and hypertension [18], and thus, often these two factors have congruent effects on outcomes. However, while disease-specific knowledge can play an important role in impacting patient outcomes, the ability to apply and learn this knowledge is a unique function of health literacy [1,19]. A study by Stellefson and colleagues showed that in chronic obstructive pulmonary disease (COPD) patients, health literacy is positively associated with both generic as well as lung-specific health-related quality of life, whereas higher COPD knowledge was associated with lower lung-specific quality of life [20], which suggests that knowledge and health literacy may not be easily equated, and as such health literacy may be a better tool to predict and impact patient-reported outcomes.

Additionally, some studies investigated patient-reported experience as a dependent measure instead of patient-reported outcomes. Even though both provide useful patient-directed information, it is important to distinguish between patient-reported experience and patient-reported outcomes. Patient-reported experience measures are reports of patient satisfaction and attitudes towards the healthcare service they are receiving [21]. They focus on a person’s general customer service experience in a healthcare setting; these can provide insight into patient safety and be used to monitor feedback but are not disease-specific. On the other hand, patient-reported outcomes can provide insight into the effectiveness of care, which may be illness-specific, from a patient’s perspective. Since our review was focused on the impact of health literacy in the context of the complex management of benign gynecological conditions, outcome-specific, rather than experience-based patient reporting tools are more useful.

Overall, the broader literature suggests that higher health literacy may be associated with better health outcomes, as systematic reviews have examined these relationships in patients with chronic kidney disease [10], hypertension [22], type 2 diabetes [23], and reproductive health and pregnancy [9]. However, studies investigating the role of health literacy and its impact in regards to patient-reported outcomes is limited and sparse. There is some evidence in other populations to suggest that higher health literacy has been associated with improved patient-reported outcomes. In COPD patients, high health literacy is related to better health-related quality of life [20]. Similarly, limited health literacy is associated with worse patient-reported outcomes, specifically lower ratings of subjective health, and depression, in patients with inflammatory bowel disease [24]. A recent 2021 study in patients with systemic lupus erythematous also found that limited health literacy is associated with worse patient-reported outcome scores, even after controlling for disease severity and damage [25].

Most of this body of work exists in populations with chronic diseases requiring lifelong medical treatment; however, one study in orthopedic surgery patients showed that patient-reported outcomes of physical function were associated with health literacy, previous surgical intervention, and socio-economic factors (age, education, education, employment) were less reliant on health literacy, based on regression analysis, as compared to the other factors [26]. These data may be more contextually relevant and translatable to our population of interest because patients with benign gynecologic disorders can often be presented with medical and surgical management options. Here, decision-making with regards to preparation for, and success of surgical interventions could be dependent on a patient’s health literacy. Thus, it is imperative to know what relationship exists between health literacy and patient-reported outcomes in patients with benign gynecological conditions, in order to understand and integrate meaningful interventions that can positively impact patient outcomes and safety.

A strength of our study is that we conducted an extensive and comprehensive literature search spanning more than 18,000 unique texts. Moreover, we were able to identify that current literature does not address the impact of health literacy on patient-reported outcomes in populations with benign gynecologic conditions. Unfortunately, since the results of our systematic review did not result in any studies that met our inclusion criteria, we were unable to address our primary and secondary objectives. Thus, we are unable to report on the relationship of health literacy and patient-reported outcomes in patients with benign gynecologic conditions, to determine the current reported prevalence of inadequate health literacy or examine demographic characteristics associated with low health literacy in this population. The authors would recommend that future studies examining patient-reported outcomes in gynecology should consider including an assessment of health literacy as part of the collected demographic information in order to be able to make robust conclusions about the impact of low health literacy on the ability of patients to complete these questionnaires as well as its impact on outcomes.

## Conclusions

This review sheds light on the gaps in the existing literature regarding the role of health literacy and its impact on patient-reported outcomes among individuals with benign gynecological conditions. Despite a comprehensive search across multiple databases, we found no published studies specifically addressing this crucial intersection.

Through our review, several recurring themes emerged, highlighting the need for further investigation. While some studies explored the relationship between health literacy and knowledge of benign gynecological conditions, others focused on patient satisfaction and experience rather than patient-reported outcomes. This differentiation underscores the importance of utilizing outcome-specific measures in understanding the true impact of health literacy in this context. Future research examining this relationship will help guide the delivery of equitable, patient-centered care in this population.

## Appendices

Final article search strategies

Note: Details have been primarily given in the order of ID, Search, Hits, unless otherwise specified

Ovid MEDLINE: Epub Ahead of Print, In-Process & Other Non-Indexed Citations, Ovid MEDLINE® Daily and Ovid MEDLINE® <1946-Present>

1          exp Gynecologic Surgical Procedures/         86231

2          exp Gynecology/ 19554

3          benign gyn?ecology.tw.    57

4          exp uterine hemorrhage/ or menorrhagia/ or metrorrhagia/      22241

5          ((abnormal or irregular or dysfunction*) adj3 uterine bleeding).tw.       3147

6          exp Leiomyoma/ 21570

7          fibroid*.tw.        7112

8          leiomyoma*.tw.  14430

9          (fibromyoma* or fibroma*).tw.      13264

10         exp Endometriosis/          22902

11         endometrio*.tw.  32387

12         exp Ovarian Cysts/          22105

13         ((mass* or cyst* or disease* or tumo?r*) adj3 (benign ovar* or benign adnexa*)).tw.       2173

14         exp Uterine Prolapse/ or exp Pelvic Organ Prolapse/  12869

15         (pelvic organ prolapse or cystocele or enterocele or apical prolapse or rectocele or anterior compartment prolapse or posterior compartment prolapse).tw.           7709

16         1 or 2 or 3 or 4 or 5 or 6 or 7 or 8 or 9 or 10 or 11 or 12 or 13 or 14 or 15           222150

17         exp Health Knowledge, Attitudes, Practice/ or exp Health Literacy/ or exp Educational Status/ or exp Health Education/ or exp Patient Education as Topic/            389098

18         exp Comprehension/        15589

19         "surveys and questionnaires"/ or self report/ 528631

20         exp Literacy/      988

21         (wide range achievement test or WRAT).tw. 447

22         (Rapid estimate of adult literacy in medicine or REALM).tw.  7956

23         (Peabody individual achievement test or PIAT).tw.    110

24         (Slosson oral reading test or SORT).tw.       13359

25         (National adult reading test or NART or AMNART).tw.         395

26         (Woodcock-Johnson and test).tw.   161

27         (Medical terminology and achievement).tw. 5

28         (literacy assessment for diabetes or adult basic education test).tw.        6

29         (Newest vital sign or NVS).tw.      812

30         (Test of health literacy in adults or TOFHLA or STOFHLA).tw.          269

31         (Short assessment of health literacy or SAHL or SAHLSA).tw.            81

32         (health literacy screening question methodologies or HLSQM).tw.       0

33         (Single-item literacy screener or SILS).tw.   1345

34         (Health literacy skills instrument or HLSI or HLSI-SF).tw.     11

35         (Medical term recognition test or METER).tw.          17229

36         (Short literacy survey or SLS).tw.   4281

37         (Brief health literacy screen or BHLS).tw.    65

38         17 or 18 or 19 or 20 or 21 or 22 or 23 or 24 or 25 or 26 or 27 or 28 or 29 or 30 or 31 or 32 or 33 or 34 or 35 or 36 or 37         904479

39         16 and 38           6871

40         exp Case Reports/ or exp Carcinoma/ or (cancer* or malignan*).tw.     4580900

41         39 not 40           5929

Embase Classic+Embase <1947 to 2021 July 09> 

1          exp Gynecologic Surgical Procedures/ or exp Gynecology/ or benign gyn?ecology.tw.     214644

2          exp uterine hemorrhage/ or menorrhagia/ or metrorrhagia/      26916

3          ((abnormal or irregular or dysfunction*) adj3 uterine bleeding).tw.       5285

4          exp Leiomyoma/ 22794

5          (fibroid* or leiomyoma* or fibromyoma* or fibroma*).tw.      48323

6          exp Endometriosis/          42295

7          endometrio*.tw.  50181

8          exp Ovarian Cysts/          44355

9          ((mass* or cyst* or disease* or tumo?r*) adj3 (benign ovar* or benign adnexa*)).tw.       3177

10         exp Uterine Prolapse/ or exp Pelvic Organ Prolapse/  24422

11         (pelvic organ prolapse or cystocele or enterocele or apical prolapse or rectocele or anterior compartment prolapse or posterior compartment prolapse).tw.            15538

12         1 or 2 or 3 or 4 or 5 or 6 or 7 or 8 or 9 or 10 or 11      374695

13         exp Health Knowledge, Attitudes, Practice/ or exp Health Literacy/ or exp Educational Status/ or exp Health Education/ or exp Patient Education as Topic/            520954

14         exp Comprehension/        32480

15         "surveys and questionnaires"/ or self report/ 858989

16         exp Literacy/      4405

17         (wide range achievement test or WRAT).tw. 639

18         (Rapid estimate of adult literacy in medicine or REALM).tw.  9529

19         (Peabody individual achievement test or PIAT).tw.    140

20         (Slosson oral reading test or SORT).tw.       19615

21         (National adult reading test or NART or AMNART).tw.         652

22         (Woodcock-Johnson and test).tw.   199

23         (Medical terminology and achievement).tw. 6

24         (literacy assessment for diabetes or adult basic education test).tw.        11

25         (Newest vital sign or NVS).tw.      1210

26         (Test of health literacy in adults or TOFHLA or STOFHLA).tw.          440

27         (Short assessment of health literacy or SAHL or SAHLSA).tw.            123

28         (health literacy screening question methodologies or HLSQM).tw.       0

29         (Single-item literacy screener or SILS).tw.   2421

30         (Health literacy skills instrument or HLSI or HLSI-SF).tw.     12

31         (Medical term recognition test or METER).tw.          25715

32         (Short literacy survey or SLS).tw.   5265

33         (Brief health literacy screen or BHLS).tw.    110

34         13 or 14 or 15 or 16 or 17 or 18 or 19 or 20 or 21 or 22 or 23 or 24 or 25 or 26 or 27 or 28 or 29 or 30 or 31 or 32 or 33         1387412

35         12 and 34           16976

36         exp Case Reports/ or exp Carcinoma/ or (cancer* or malignan*).tw.     3934367

37         35 not 36           14715

The Cochrane Library

#1         (benign gyn?ecology):ti,ab,kw       182

#2         MeSH descriptor: [Gynecologic Surgical Procedures] explode all trees  4461

#3         MeSH descriptor: [Gynecology] explode all trees      160

#4         MeSH descriptor: [Uterine Hemorrhage] explode all trees       1839

#5         MeSH descriptor: [Menorrhagia] explode all trees     399

#6         MeSH descriptor: [Metrorrhagia] explode all trees     146

#7         (((abnormal or irregular or dysfunction*) Near/3 uterine bleeding)):ti,ab,kw       646

#8         MeSH descriptor: [Leiomyoma] explode all trees      710

#9         ((fibromyoma* or fibroma*)):ti,ab,kw         207

#10       MeSH descriptor: [Endometriosis] explode all trees   879

#11       (endometrio*):ti,ab,kw     3022

#12       MeSH descriptor: [Ovarian Cysts] explode all trees   1714

#13       (((mass* or cyst* or disease* or tumo?r*) Near/3 (benign ovar* or benign adnexa*))):ti,ab,kw      4649

#14       MeSH descriptor: [Uterine Prolapse] explode all trees 210

#15       MeSH descriptor: [Pelvic Organ Prolapse] explode all trees     601

#16       (("pelvic organ prolapse" or "cystocele" or "enterocele" or "apical prolapse" or "rectocele" or "anterior compartment prolapse" or "posterior compartment prolapse")):ti,ab,kw         1769

#17       {OR #1-#16}      17132

#18       MeSH descriptor: [Health Knowledge, Attitudes, Practice] explode all trees       6104

#19       MeSH descriptor: [Health Literacy] explode all trees 402

#20       MeSH descriptor: [Educational Status] explode all trees         1490

#21       MeSH descriptor: [Health Education] explode all trees           20556

#22       MeSH descriptor: [Patient Education as Topic] explode all trees          9105

#23       MeSH descriptor: [Comprehension] explode all trees 624

#24       MeSH descriptor: [Self Report] explode all trees       2416

#25       MeSH descriptor: [Literacy] explode all trees           34

#26       (("wide range achievement test" or WRAT)):ti,ab,kw 58

#27       (("Rapid estimate of adult literacy in medicine" or REALM)):ti,ab,kw   187

#28       (("Peabody individual achievement test" or PIAT)):ti,ab,kw    4

#29       (("Slosson oral reading test" or SORT)):ti,ab,kw       668

#30       (("National adult reading test" or NART or AMNART)):ti,ab,kw         64

#31       (("Woodcock-Johnson" and test)):ti,ab,kw   21

#32       (("Medical terminology" and achievement)):ti,ab,kw  0

#33       (("Short literacy survey" or SLS)):ti,ab,kw   458

#34       (("Brief health literacy screen" or BHLS)):ti,ab,kw    4

#35       (("literacy assessment for diabetes" or "adult basic education test")):ti,ab,kw      1

#36       ("Newest vital sign" or NVS):ti,ab,kw OR ("Short assessment of health literacy" or SAHL or SAHLSA):ti,ab,kw OR ("health literacy screening question methodologies" or HLSQM):ti,ab,kw AND ("Single-item literacy screener" or SILS):ti,ab,kw AND ("Health literacy skills instrument" or HLSI or HLSI-SF):ti,ab,kw      105

#37       ("Medical term recognition test" or METER):ti,ab,kw 5223

#38       {OR #18-#37}    33320

#39       {AND #17, #38} 137

#40       MeSH descriptor: [Case Reports] explode all trees     0

#41       MeSH descriptor: [Carcinoma] explode all trees        13990

#42       ((cancer* or malignan*)):ti,ab,kw   181797

#43       {OR #40-#42}    186475

#44       #39 NOT #43     94

*Web of Science* ((TS=((Gyn$ecologic Surgical Procedures) OR (Gyn$ecology) OR (benign gyn$ecology) OR (uterine hemorrhage) OR (menorrhagia) OR (metrorrhagia) OR (abnormal uterine bleed*) OR (irregular uterine bleed*) OR (dysfunction* uterine bleed*) OR (leiomyoma) OR (fibroid*) OR (fibromyoma) OR (fibroma) OR (Endometriosis) OR (ovar* cyst*) OR (((benign ovar*) or (benign adnexa*)) AND ((mass*) OR (cyst*) OR (disease*) OR (tumo$r*))) OR (uterine prolapse*) OR (pelvic organ prolapse*) OR (cystocele) OR (enterocele) OR (apical prolapse) OR (rectocele) OR (anterior compartment prolapse) OR (posterior compartment prolapse))) 

AND 

(TS=((Health literacy) OR (Literacy) OR (Comprehension) OR (Health knowledge) OR (Education* status) OR (Health education) OR (Patient* education*) OR ("wide range achievement test") OR (WRAT) OR ("Rapid estimate of adult literacy in medicine") OR (REALM) OR ("Peabody individual achievement test") OR (PIAT) OR ("Slosson oral reading test") OR (SORT) OR ("National adult reading test") OR (NART) OR (AMNART) OR ("Woodcock-Johnson" AND test) OR ("Medical terminology" AND "achievement") OR ("literacy assessment for diabetes") OR ("adult basic education test") OR ("Newest vital sign") OR (NVS) OR ("Test of health literacy in adults") OR (TOFHLA) OR (STOFHLA) OR ("Short assessment of health literacy") OR (SAHL) OR (SAHLSA) OR  ("health literacy screening question methodologies") OR (HLSQM) OR ("Single-item literacy screener" OR (SILS) OR ("Health literacy skills instrument") OR (HLSI) OR (HLSI-SF) OR ("Medical term recognition test") OR (METER) OR ("Short literacy survey") OR (SLS) OR (Brief health literacy screen) OR (BHLS))))) 

NOT

(TS=((Carcinoma*) OR (Cancer*) OR (Malignan*) OR (Case report*)))

2925 results

Clinicaltrials.gov

Searched for “Health Literacy” as a condition or disease term. And then applied filters of “Completed”, “Female”, “Adult(18-64)” and “Older adult (65+)” on search. 

141 results

PUBMED

Search number, Query, Sort By, Results 

19 

((((((((((((((((((Gynecologic Surgical Procedures[MeSH Terms]) OR (Gynecology[MeSH Terms])) OR (uterine hemorrhage[MeSH Terms])) OR (menorrhagia[MeSH Terms])) OR (metrorrhagia[MeSH Terms])) OR (leiomyoma[MeSH Terms])) OR (endometriosis[MeSH Terms])) OR (ovarian cysts[MeSH Terms])) OR (uterine prolapse[MeSH Terms])) OR (pelvic organ prolapse[MeSH Terms])) OR (benign gynecology[Title/Abstract])) OR (((abnormal uterine bleeding[Title/Abstract]) OR (irregular uterine bleeding[Title/Abstract])) OR (dysfunction* uterine bleeding[Title/Abstract]))) OR ((fibromyoma[Title/Abstract]) OR (fibroma[Title/Abstract]))) OR (endometrio*[Title/Abstract])) OR (((benign ovar* n3 mass*[Title/Abstract]) OR (benign ovar* n3 cyst*[Title/Abstract])) OR (benign ovar* n3 tumor*[Title/Abstract]))) OR (((benign adnexa* n3 mass*[Title/Abstract]) OR (benign adnexa* n3 cyst*[Title/Abstract])) OR (benign adnexa* n3 tumor*[Title/Abstract]))) OR ((((((((pelvic organ prolapse[Title/Abstract]) ) OR (cystocele[Title/Abstract])) OR (enterocele[Title/Abstract])) OR (rectocele[Title/Abstract])) OR ("apical prolapse"[Title/Abstract])) OR ("anterior compartment prolapse"[Title/Abstract])) OR ("posterior compartment prolapse"[Title/Abstract]))) AND (((((((((health knowledge, attitudes, practice[MeSH Terms]) OR (health literacy[MeSH Terms])) OR (educational status[MeSH Terms])) OR (health education[MeSH Terms])) OR (patient education as topic[MeSH Terms])) OR (comprehension[MeSH Terms])) OR (self report[MeSH Terms])) OR (Literacy[MeSH Terms])) OR ((((((((((((((((((((((((((((((("wide range achievement test"[Title/Abstract]) OR (WRAT[Title/Abstract])) OR ("Rapid estimate of adult literacy in medicine"[Title/Abstract])) OR (REALM[Title/Abstract])) OR ("Peabody individual achievement test"[Title/Abstract])) OR (PIAT[Title/Abstract])) OR ("Slosson oral reading test"[Title/Abstract])) OR (SORT[Title/Abstract])) OR ("National adult reading test"[Title/Abstract])) OR (NART[Title/Abstract])) OR (AMNART[Title/Abstract])) OR ("Woodcock-Johnson"[Title/Abstract])) OR ("Medical terminology"[Title/Abstract] AND achievement[Title/Abstract])) OR ("Short literacy survey"[Title/Abstract])) OR (SLS[Title/Abstract])) OR ("Brief health literacy screen"[Title/Abstract])) OR (BHLS[Title/Abstract])) OR ("Newest vital sign"[Title/Abstract])) OR (NVS[Title/Abstract])) OR ("Short assessment of health literacy"[Title/Abstract])) OR (SAHL[Title/Abstract])) OR (SAHLSA[Title/Abstract])) OR ("health literacy screening question methodologies"[Title/Abstract])) OR (HLSQM[Title/Abstract])) OR ("Single-item literacy screener"[Title/Abstract])) OR (SILS[Title/Abstract])) OR ("Health literacy skills instrument"[Title/Abstract])) OR (HLSI[Title/Abstract])) OR (HLSI-SF[Title/Abstract])) OR ("Medical term recognition test"[Title/Abstract])) OR (METER[Title/Abstract])))) NOT ((((Cancer[MeSH Terms]) OR (Carcinoma[MeSH Terms])) OR (case reports[MeSH Terms])) OR ((Cancer*[Title/Abstract]) OR (malignan*[Title/Abstract]))) 

15 NOT 18 

1,896 

18 

(((Cancer[MeSH Terms]) OR (Carcinoma[MeSH Terms])) OR (case reports[MeSH Terms])) OR ((Cancer*[Title/Abstract]) OR (malignan*[Title/Abstract])) 

16 OR 17 

4,167,531 

17 

(Cancer*[Title/Abstract]) OR (malignan*[Title/Abstract]) 

2,339,277 

16 

((Cancer[MeSH Terms]) OR (Carcinoma[MeSH Terms])) OR (case reports[MeSH Terms]) 

3,501,617 

15 

(((((((((((((((((Gynecologic Surgical Procedures[MeSH Terms]) OR (Gynecology[MeSH Terms])) OR (uterine hemorrhage[MeSH Terms])) OR (menorrhagia[MeSH Terms])) OR (metrorrhagia[MeSH Terms])) OR (leiomyoma[MeSH Terms])) OR (endometriosis[MeSH Terms])) OR (ovarian cysts[MeSH Terms])) OR (uterine prolapse[MeSH Terms])) OR (pelvic organ prolapse[MeSH Terms])) OR (benign gynecology[Title/Abstract])) OR (((abnormal uterine bleeding[Title/Abstract]) OR (irregular uterine bleeding[Title/Abstract])) OR (dysfunction* uterine bleeding[Title/Abstract]))) OR ((fibromyoma[Title/Abstract]) OR (fibroma[Title/Abstract]))) OR (endometrio*[Title/Abstract])) OR (((benign ovar* n3 mass*[Title/Abstract]) OR (benign ovar* n3 cyst*[Title/Abstract])) OR (benign ovar* n3 tumor*[Title/Abstract]))) OR (((benign adnexa* n3 mass*[Title/Abstract]) OR (benign adnexa* n3 cyst*[Title/Abstract])) OR (benign adnexa* n3 tumor*[Title/Abstract]))) OR ((((((((pelvic organ prolapse[Title/Abstract]) ) OR (cystocele[Title/Abstract])) OR (enterocele[Title/Abstract])) OR (rectocele[Title/Abstract])) OR ("apical prolapse"[Title/Abstract])) OR ("anterior compartment prolapse"[Title/Abstract])) OR ("posterior compartment prolapse"[Title/Abstract]))) AND (((((((((health knowledge, attitudes, practice[MeSH Terms]) OR (health literacy[MeSH Terms])) OR (educational status[MeSH Terms])) OR (health education[MeSH Terms])) OR (patient education as topic[MeSH Terms])) OR (comprehension[MeSH Terms])) OR (self report[MeSH Terms])) OR (Literacy[MeSH Terms])) OR ((((((((((((((((((((((((((((((("wide range achievement test"[Title/Abstract]) OR (WRAT[Title/Abstract])) OR ("Rapid estimate of adult literacy in medicine"[Title/Abstract])) OR (REALM[Title/Abstract])) OR ("Peabody individual achievement test"[Title/Abstract])) OR (PIAT[Title/Abstract])) OR ("Slosson oral reading test"[Title/Abstract])) OR (SORT[Title/Abstract])) OR ("National adult reading test"[Title/Abstract])) OR (NART[Title/Abstract])) OR (AMNART[Title/Abstract])) OR ("Woodcock-Johnson"[Title/Abstract])) OR ("Medical terminology"[Title/Abstract] AND achievement[Title/Abstract])) OR ("Short literacy survey"[Title/Abstract])) OR (SLS[Title/Abstract])) OR ("Brief health literacy screen"[Title/Abstract])) OR (BHLS[Title/Abstract])) OR ("Newest vital sign"[Title/Abstract])) OR (NVS[Title/Abstract])) OR ("Short assessment of health literacy"[Title/Abstract])) OR (SAHL[Title/Abstract])) OR (SAHLSA[Title/Abstract])) OR ("health literacy screening question methodologies"[Title/Abstract])) OR (HLSQM[Title/Abstract])) OR ("Single-item literacy screener"[Title/Abstract])) OR (SILS[Title/Abstract])) OR ("Health literacy skills instrument"[Title/Abstract])) OR (HLSI[Title/Abstract])) OR (HLSI-SF[Title/Abstract])) OR ("Medical term recognition test"[Title/Abstract])) OR (METER[Title/Abstract]))) 

#11 AND #14 

2,536 

14 

((((((((health knowledge, attitudes, practice[MeSH Terms]) OR (health literacy[MeSH Terms])) OR (educational status[MeSH Terms])) OR (health education[MeSH Terms])) OR (patient education as topic[MeSH Terms])) OR (comprehension[MeSH Terms])) OR (self report[MeSH Terms])) OR (Literacy[MeSH Terms])) OR ((((((((((((((((((((((((((((((("wide range achievement test"[Title/Abstract]) OR (WRAT[Title/Abstract])) OR ("Rapid estimate of adult literacy in medicine"[Title/Abstract])) OR (REALM[Title/Abstract])) OR ("Peabody individual achievement test"[Title/Abstract])) OR (PIAT[Title/Abstract])) OR ("Slosson oral reading test"[Title/Abstract])) OR (SORT[Title/Abstract])) OR ("National adult reading test"[Title/Abstract])) OR (NART[Title/Abstract])) OR (AMNART[Title/Abstract])) OR ("Woodcock-Johnson"[Title/Abstract])) OR ("Medical terminology"[Title/Abstract] AND achievement[Title/Abstract])) OR ("Short literacy survey"[Title/Abstract])) OR (SLS[Title/Abstract])) OR ("Brief health literacy screen"[Title/Abstract])) OR (BHLS[Title/Abstract])) OR ("Newest vital sign"[Title/Abstract])) OR (NVS[Title/Abstract])) OR ("Short assessment of health literacy"[Title/Abstract])) OR (SAHL[Title/Abstract])) OR (SAHLSA[Title/Abstract])) OR ("health literacy screening question methodologies"[Title/Abstract])) OR (HLSQM[Title/Abstract])) OR ("Single-item literacy screener"[Title/Abstract])) OR (SILS[Title/Abstract])) OR ("Health literacy skills instrument"[Title/Abstract])) OR (HLSI[Title/Abstract])) OR (HLSI-SF[Title/Abstract])) OR ("Medical term recognition test"[Title/Abstract])) OR (METER[Title/Abstract])) 

#12 OR #13 

480,850 

13 

(((((((((((((((((((((((((((((("wide range achievement test"[Title/Abstract]) OR (WRAT[Title/Abstract])) OR ("Rapid estimate of adult literacy in medicine"[Title/Abstract])) OR (REALM[Title/Abstract])) OR ("Peabody individual achievement test"[Title/Abstract])) OR (PIAT[Title/Abstract])) OR ("Slosson oral reading test"[Title/Abstract])) OR (SORT[Title/Abstract])) OR ("National adult reading test"[Title/Abstract])) OR (NART[Title/Abstract])) OR (AMNART[Title/Abstract])) OR ("Woodcock-Johnson"[Title/Abstract])) OR ("Medical terminology"[Title/Abstract] AND achievement[Title/Abstract])) OR ("Short literacy survey"[Title/Abstract])) OR (SLS[Title/Abstract])) OR ("Brief health literacy screen"[Title/Abstract])) OR (BHLS[Title/Abstract])) OR ("Newest vital sign"[Title/Abstract])) OR (NVS[Title/Abstract])) OR ("Short assessment of health literacy"[Title/Abstract])) OR (SAHL[Title/Abstract])) OR (SAHLSA[Title/Abstract])) OR ("health literacy screening question methodologies"[Title/Abstract])) OR (HLSQM[Title/Abstract])) OR ("Single-item literacy screener"[Title/Abstract])) OR (SILS[Title/Abstract])) OR ("Health literacy skills instrument"[Title/Abstract])) OR (HLSI[Title/Abstract])) OR (HLSI-SF[Title/Abstract])) OR ("Medical term recognition test"[Title/Abstract])) OR (METER[Title/Abstract]) 

46,716 

12 

(((((((health knowledge, attitudes, practice[MeSH Terms]) OR (health literacy[MeSH Terms])) OR (educational status[MeSH Terms])) OR (health education[MeSH Terms])) OR (patient education as topic[MeSH Terms])) OR (comprehension[MeSH Terms])) OR (self report[MeSH Terms])) OR (Literacy[MeSH Terms]) 

435,775 

11 

((((((((((((((((Gynecologic Surgical Procedures[MeSH Terms]) OR (Gynecology[MeSH Terms])) OR (uterine hemorrhage[MeSH Terms])) OR (menorrhagia[MeSH Terms])) OR (metrorrhagia[MeSH Terms])) OR (leiomyoma[MeSH Terms])) OR (endometriosis[MeSH Terms])) OR (ovarian cysts[MeSH Terms])) OR (uterine prolapse[MeSH Terms])) OR (pelvic organ prolapse[MeSH Terms])) OR (benign gynecology[Title/Abstract])) OR (((abnormal uterine bleeding[Title/Abstract]) OR (irregular uterine bleeding[Title/Abstract])) OR (dysfunction* uterine bleeding[Title/Abstract]))) OR ((fibromyoma[Title/Abstract]) OR (fibroma[Title/Abstract]))) OR (endometrio*[Title/Abstract])) OR (((benign ovar* n3 mass*[Title/Abstract]) OR (benign ovar* n3 cyst*[Title/Abstract])) OR (benign ovar* n3 tumor*[Title/Abstract]))) OR (((benign adnexa* n3 mass*[Title/Abstract]) OR (benign adnexa* n3 cyst*[Title/Abstract])) OR (benign adnexa* n3 tumor*[Title/Abstract]))) OR ((((((((pelvic organ prolapse[Title/Abstract]) ) OR (cystocele[Title/Abstract])) OR (enterocele[Title/Abstract])) OR (rectocele[Title/Abstract])) OR ("apical prolapse"[Title/Abstract])) OR ("anterior compartment prolapse"[Title/Abstract])) OR ("posterior compartment prolapse"[Title/Abstract])) 

OR #1-#10 

211,573 

10 

(((((((pelvic organ prolapse[Title/Abstract]) ) OR (cystocele[Title/Abstract])) OR (enterocele[Title/Abstract])) OR (rectocele[Title/Abstract])) OR ("apical prolapse"[Title/Abstract])) OR ("anterior compartment prolapse"[Title/Abstract])) OR ("posterior compartment prolapse"[Title/Abstract]) 

7,999 

9 

((benign adnexa* n3 mass*[Title/Abstract]) OR (benign adnexa* n3 cyst*[Title/Abstract])) OR (benign adnexa* n3 tumor*[Title/Abstract]) 

12 

8 

((benign ovar* n3 mass*[Title/Abstract]) OR (benign ovar* n3 cyst*[Title/Abstract])) OR (benign ovar* n3 tumor*[Title/Abstract]) 

34 

5 

endometrio*[Title/Abstract] 

32,908 

4 

(fibromyoma[Title/Abstract]) OR (fibroma[Title/Abstract]) 

8,669 

3 

((abnormal uterine bleeding[Title/Abstract]) OR (irregular uterine bleeding[Title/Abstract])) OR (dysfunction* uterine bleeding[Title/Abstract]) 

3,249 

2 

benign gynecology[Title/Abstract] 

50 

1 

(((((((((Gynecologic Surgical Procedures[MeSH Terms]) OR (Gynecology[MeSH Terms])) OR (uterine hemorrhage[MeSH Terms])) OR (menorrhagia[MeSH Terms])) OR (metrorrhagia[MeSH Terms])) OR (leiomyoma[MeSH Terms])) OR (endometriosis[MeSH Terms])) OR (ovarian cysts[MeSH Terms])) OR (uterine prolapse[MeSH Terms])) OR (pelvic organ prolapse[MeSH Terms]) 

188,359 
